# Mucosal Eosinophil Abundance in Non-Inflamed Colonic Tissue Is Associated with Response to Vedolizumab Induction Therapy in Inflammatory Bowel Disease

**DOI:** 10.3390/jcm11144141

**Published:** 2022-07-16

**Authors:** Ruben Y. Gabriëls, Arno R. Bourgonje, Julius Z. H. von Martels, Tjasso Blokzijl, Rinse K. Weersma, Kevin Galinsky, Julius Juarez, Klaas Nico Faber, Gursah Kats-Ugurlu, Gerard Dijkstra

**Affiliations:** 1 Department of Gastroenterology and Hepatology, University Medical Center Groningen, University of Groningen, Hanzeplein 1, 9713 GZ Groningen, The Netherlands; a.r.bourgonje@umcg.nl (A.R.B.); j.z.h.von.martels@umcg.nl (J.Z.H.v.M.); t.blokzijl@umcg.nl (T.B.); r.k.weersma@umcg.nl (R.K.W.); k.n.faber@umcg.nl (K.N.F.); gerard.dijkstra@umcg.nl (G.D.); 2 Gastroenterology Drug Discovery Unit, Takeda Pharmaceuticals, Cambridge, MA 02139, USA; kevin.galinsky@takeda.com (K.G.); julius.juarez@takeda.com (J.J.); 3 Department of Pathology and Medical Biology, University Medical Center Groningen, University of Groningen, Hanzeplein 1, 9713 GZ Groningen, The Netherlands; g.kats-ugurlu@umcg.nl

**Keywords:** inflammatory bowel disease, vedolizumab, eosinophil, eotaxin-1

## Abstract

Vedolizumab is used as a treatment for patients with inflammatory bowel disease (IBD), but induction therapy leads to clinical response and remission in approximately 55% and 30% of patients with IBD, respectively. In this study, we aimed to explore the predictive value of mucosal eosinophils and serum eotaxin-1 regarding response to vedolizumab induction therapy. Eighty-four (84) patients with IBD (37 Crohn’s disease [CD], 47 ulcerative colitis [UC]) were included. For 24 patients with IBD, histopathology was assessed for eosinophil counts in non-inflamed colonic tissue prior to vedolizumab treatment. For 64 patients with IBD, serum eotaxin-1 levels were quantified prior to (baseline) and during vedolizumab treatment. Serum samples of 100 patients with IBD (34 CD, 66 UC) from the GEMINI 1 and 2 trials were used for external validation. Baseline mucosal eosinophil numbers in non-inflamed colonic tissue were significantly higher in responders to vedolizumab induction therapy when compared to primary non-responders (69 [34–138] vs. 24 [18–28] eosinophils/high-power field, respectively, *p* < 0.01). Baseline serum eotaxin-1 levels in the discovery cohort were significantly elevated in responders, compared to primary non-responders (0.33 [0.23–0.44] vs. 0.20 [0.16–0.29] ng/mL, *p* < 0.01). Prediction models based on mucosal eosinophil counts and serum eotaxin-1 showed an area under the curve (AUC) of 0.90 and 0.79, respectively. However, the predictive capacity of baseline serum eotaxin-1 levels could not be validated in the GEMINI cohort. Mucosal eosinophil abundance in non-inflamed colonic tissue was associated with response to vedolizumab induction therapy in patients with IBD. Future studies are warranted to further validate the potential value of mucosal eosinophils and serum eotaxin-1 as biomarkers for response to vedolizumab therapy.

## 1. Introduction

Crohn’s disease (CD) and ulcerative colitis (UC) are chronic inflammatory diseases of the gastrointestinal (GI) tract, collectively referred to as inflammatory bowel diseases (IBD) [1]. Current medical treatment for IBD includes aminosalicylates, corticosteroids, immunomodulators and TNF-α antagonists [2,3]. Vedolizumab is a humanized monoclonal antibody directed against the α4β7 integrin dimer and blocks the migration of several immune cells across the endothelium [4]. Vedolizumab has shown efficacy, safety and tolerability as a treatment for patients with IBD [5,6]. In randomized controlled trials, vedolizumab continued to exhibit reasonable performance, which led to its approval for the treatment of adult patients with moderate-to-severe active IBD. It may be used as a first-line biologic agent or in patients who are refractory to TNF-α antagonist therapy [7,8]. Additionally, vedolizumab therapy increases quality-adjusted-life years (QALY) and is also a cost-effective treatment for IBD [9]. Despite its long-term efficacy, vedolizumab induction therapy (traditionally measured at 6 or 14 weeks after therapy initiation) only shows clinical response and clinical remission in 56–58% vs. 30% of patients with CD and 43–56% vs. 32% of patients with UC, respectively [10]. Real-life IBD cohorts show similar efficacy results concerning response to vedolizumab induction therapy [11,12,13,14]. Approximately 20% of all vedolizumab-treated patients with IBD will eventually discontinue treatment due to loss of response, as observed in extended follow-up trials [15,16].

Peripheral blood eosinophilia is associated with severe IBD disease activity [17]. Eosinophils accumulate in the gut mucosa of patients with IBD with active disease and may play a role in its pathogenesis. Eotaxin-1 (CCL11) is a selective chemoattractant that triggers the activation and mobilization of eosinophils to the lamina propria of the gut. Vedolizumab binds to several types of immune cells and might reduce eosinophilic trafficking to the intestine [4]. Therefore, gut eosinophil abundance might predict therapy response to vedolizumab in IBD [18,19,20,21]. In this study, we aimed to analyze the relation between the number of mucosal eosinophils, peripheral eosinophils and serum eotaxin-1 levels in patients with aiming to predict response to vedolizumab induction therapy.

## 2. Materials and Methods

### 2.1. Study Population and Data Collection

This retrospective cohort study included patients with IBD treated in the past five years at the IBD centre of the University Medical Centre Groningen (UMCG). All included patients (in total 84 patients with IBD: 37 with Crohn’s disease [CD], and 47 with ulcerative colitis [UC]) had an established diagnosis of IBD existing for at least 1 year, either Crohn’s disease (CD) or ulcerative colitis (UC), and were treated with vedolizumab induction therapy. In a subset of 24 of these patients (9 having CD, 15 having UC) histopathological data were analyzed for eosinophilic granulocyte counts in high-power fields (hpf) of non-inflamed parts of ascending colonic tissue prior to vedolizumab treatment.

In another subset of patients, consisting of 64 patients with IBD (28 CD, 36 UC), serum eotaxin-1 levels were quantified prior to vedolizumab treatment. All recruited patients were treated with 300 mg vedolizumab intravenously at standardized clinical visits at weeks 0, 2, 6 and 14, the latter being considered the end of induction therapy. Exclusion criteria included age < 18 years or patients with comorbidities causing significant changes in blood leukocyte distributions (e.g., HIV or lymphoproliferative disorders). Age, gender, body-mass index (BMI), smoking status, Montreal classification, current medication use (aminosalicylates, thiopurines, methotrexate, TNF-α--antagonists), previous anti-TNF-α therapy and surgical history (ileocecal resection, colectomy) were retrieved from medical records. At each clinical visit, hemoglobin levels, C-reactive protein (CRP) levels, erythrocyte sedimentation rates (ESR), white blood cell counts (WBC), thrombocyte counts, and eosinophil counts were determined.

Additionally, serum samples of 100 patients with IBD (66 UC, 34 CD) derived from the GEMINI 1 and 2 pivotal clinical trials, were analyzed for serum eotaxin-1 levels at baseline and at week 6 vedolizumab induction theapy to externally validate our findings [7,8]. Pairs of vedolizumab-treated week 6 responders and non-responders to vedolizumab induction therapy were selected from each GEMINI study, matching samples on baseline fecal calprotectin levels and CDAI (for CD samples) or Mayo (for UC samples) scores, resulting in 17 pairs of patients with CD and 33 pairs of patients with UC that were selected. An overview of the study design can be found in Figure S1.

### 2.2. Definition of Study Outcomes

The primary study outcome was defined as clinical response or remission after vedolizumab induction therapy at week 14. Clinical response was defined as a decrease of at least 3 points in the Harvey-Bradshaw Index (HBI) for CD or Simple Clinical Colitis Activity Index (SCCAI) for UC from baseline or by assessment of the treating physician [22,23]. Clinical remission was defined as HBI ≤ 3 for CD and SCCAI ≤ 2.5 for UC or by the physician’s global assessment (PGA). Primary non-responders were defined as patients whose therapy was ceased before the end of induction therapy or patients that did not meet the aforementioned clinical response or remission criteria.

Clinical response to vedolizumab induction therapy in the GEMINI 1 and 2 trials, in which data and serum samples were used for external validation of the analyses in relation to the serum eotaxin-1 biomarker, was assessed after 6 weeks of induction therapy, as well as after 14 and 52 weeks of maintenance therapy.

### 2.3. Histopathological Data

We retrospectively collected hematoxylin and eosin (HE)-slides of formalin-fixed paraffine-embedded pre-treatment colonic biopsies from 24 patients taken within a maximum window of 90 days from baseline. Initially two observers (RYG, GKU) pathologically evaluated the biopsies of inflamed and non-inflamed tissue independently. The preliminary results showed that agreement on inflamed tissue could often not be achieved during the manual counting of the eosinophils due to group formation and mixing with other inflammatory cells, mainly neutrophils. Due to heavy inflammation, it was difficult to choose the right hpf with 400× magnification. Another issue encountered was the variability of the distribution of the eosinophils within different parts of the lower GI tract (ileum, ascending colon, transverse colon, descending colon, sigmoid and rectum) [24].

In contrast to inflamed biopsies, manual eosinophilic counts were reliable with a high level of consensus on the chosen hpf in non-inflamed parts of the collected biopsies. To avoid location-associated differences in baseline eosinophil counts, we proceeded using biopsies from the same location from all patients. Non-inflamed tissue from the ascending colon was available from biopsies of all patients of whom biopsies were available (24 patients).

All selected HE-stained slides were digitalized with IntelliSite Ultra FastScanner (Philips, Eindhoven, The Netherlands). First, eyeballing was performed to identify hotspots, areas containing the highest density of eosinophils. Second, slides were overviewed at low magnification and five high-power fields (hpf’s, area 0.24 mm^2^) were selected for each patient where there was an increment in the presence of eosinophils (“hotspots”). This method has been previously published and is clinically used for the diagnosis of eosinophilic esophagitis [25].

The trained clinical researcher (RYG) counted all eosinophils in all 5 hpf’s from each slide, amounting to a total of twenty-four slides. The pathologist (GK-U) blindly and independently counted eosinophils within the same areas. Pre-treatment eosinophil counts were documented as the maximum number of eosinophils per HPF.

### 2.4. Measurement of Serum Eotaxin-1 Levels

Measurements of serum eotaxin-1 levels were performed as previously described [26]. In short, serum samples from 64 patients with IBD at different time points were collected and stored in 1 mL aliquots in the freezer (−80 °C). Prior to analysis, samples were quickly centrifuged to remove remaining particulates. Measurements of serum eotaxin-1 were implemented using electrochemiluminescence (ECL) multiplex assays (Meso Scale Discovery (MSD^®^), Meso Scale Diagnostics, Rockville, MD). ECL signals were fitted to a 4-parameter logistic model with 1/y^2^ weighting, ensuring a broad and dynamic range of molecule detection. Serum concentrations of eotaxin-1 were determined by using calibration curves to which the ECL signals were back-fitted. Final concentrations were calculated using the MSD Discovery Workbench analysis software^®^. All concentrations were above the lower limit of detection (LLoD, for eotaxin-1: median 3.26 pg/mL, range: 2.41–5.13 pg/mL).

### 2.5. Statistics

Baseline demographic and clinical characteristics were presented as means ± standard deviations (SD), medians with interquartile ranges (IQR) or proportions *n* with corresponding percentages (%). Assessment of normality of continuous variables was performed by visual inspection of normal probability plots and histograms. Differences in demographic, clinical and laboratory data were compared using independent sample *t*-tests, Mann–Whitney *U*-tests, chi-square tests, or Fisher’s exact tests, depending on normality and type of variable. Mucosal eosinophil counts and serum eotaxin-1 levels were presented as median [IQR], and differences between groups were tested non-parametrically using Mann–Whitney *U*-tests. Correlations between different parameters were calculated using Pearson’s correlation coefficients. Univariable logistic regression analysis (method: enter) was performed to identify predictors for clinical response or remission to vedolizumab induction therapy at week 14. Non-normally distributed (biomarker) variables were ^2^log-transformed to facilitate results interpretation (per doubling). Subsequently, multivariable logistic regression analysis was performed using forced entry of variables to allow for covariate adjustment. Significant results (pre-selection threshold: *p*-value ≤ 0.05) from univariable analysis were incorporated into multivariable logistic regression analysis. Biomarkers were adjusted for demographic and clinical covariates, which consisted of (1) variables that were significantly associated with the study outcome (clinical response) based on univariable analysis (using the pre-selection threshold) and (2) variables that were considered clinically relevant. All logistic regression analyses were performed for the total IBD cohort, and for patients with CD and UC separately. Receiver operating characteristics (ROC) statistics with the area under the ROC curve (AUC) as overall measure of fit and corresponding 95% confidence intervals (CIs) were used to assess discriminative ability of the predictive biomarkers with regard to the outcome. ROC curves and AUCs were calculated using the non-parametric, tie-corrected trapezoidal approximation method. Discriminative performance of adjusted models was determined by ROC estimation of combined predicted probabilities from logistic regression. In addition, all model AUCs were internally validated using *k*-fold cross-validation (*k* = 10). In this procedure, the dataset was randomly partitioned into *k* equally sized folds, where each fold was then left out (10% of cases) while the model was fitted to the remaining *k* − 1 folds (90% of cases, training set) and predictions were obtained for the left-out part (test set). This procedure was repeated 10 times, where AUCs from each fold were averaged and bootstrapped (*n* = 500 iterations) to achieve statistical inference, resulting in a cross-validated AUC (cv-AUC). Optimal cut-off (*c*) thresholds were determined by equally maximizing sensitivity and specificity to compute the Youden’s *J* statistic, defined as *J* = max_c_ {sensitivity(*c*) + specificity(*c*) − 1}. Data were analyzed using SPSS Statistics 25.0 software package, the Python programming language (v.3.8.5, Python Software Foundation, Wilmington, DE, USA, https://python.org, accessed on 19 August 2021), using the *pandas* (v.1.2.3), *numpy* (v.1.20.0), and *statsmodels* (v.0.12.2) modules, and the R programming language (v.4.0.2, R Foundation for Statistical Computing, Vienna, Austria, https://www.r-project.org/, accessed on 19 August 2021). Data visualization was performed using *seaborn* (v.0.11.1) and *matplotlib* (v.3.4.1) packages in Python. Two-tailed *p*-values ≤ 0.05 were considered statistically significant.

### 2.6. External Validation of the Serum Eotaxin-1 Biomarker

Wilcoxon signed-rank tests were used to compare eotaxin-1 levels in Week 6 responder/non-responder pairs from the GEMINI trials [7,8]. A mixed-effects logistic regression model was fitted with response to therapy at week 14 or week 52 as outcome variables and eotaxin-1 concentrations as predictor while adjusting for week 6 response, randomization to the maintenance arm, baseline Mayo or CDAI scores, baseline fecal calprotectin, age, and sex as covariates.

### 2.7. Ethical Considerations

This study was approved by the Institutional Review Board (IRB) of the UMCG (registered as no. 2008/338). All patients provided written informed consent for their participation in the study and use of their data and biomaterials. The study has been performed in accordance with the principles of the Declaration of Helsinki (2013).

## 3. Results

### 3.1. Study Population Characteristics

An overview and comparison of baseline demographic and clinical characteristics of the discovery cohort population are presented in Table 1. The total study population consisted of 84 patients with IBD receiving vedolizumab induction therapy, of which 38 patients (45%) initially showed either response (14%) or remission (31%) at week 14, whereas 46 patients (55%) were considered primary non-responders. There was no significant difference in response or remission rates among patients with CD or UC (34% vs. 66%, respectively, *p* = 0.10). Seventy-seven (91%) of the patients had a prior failure with biological therapy. Patients who initially responded had a mean age of 44 ± 16 years (22 males (58%) and consisted of 16 females (42%)), whilst patients who were primary non-responders had a mean age of 43 ± 15 years (15 males (33%) and consisted of 31 females (67%), age, *p* = 0.95; sex, *p* < 0.05)). In the total IBD cohort, and for CD separately, serum levels of C-reactive protein (CRP) were significantly lower at baseline in patients who eventually responded to vedolizumab induction therapy, as compared to primary non-responders (IBD: 3.4 [1.3;9.5] vs. 7.0 [3.2;16.8] mg/L, respectively, *p* < 0.05, Table 1).

### 3.2. Eosinophil Counts and Vedolizumab Induction Therapy Response

**Figure 1:**
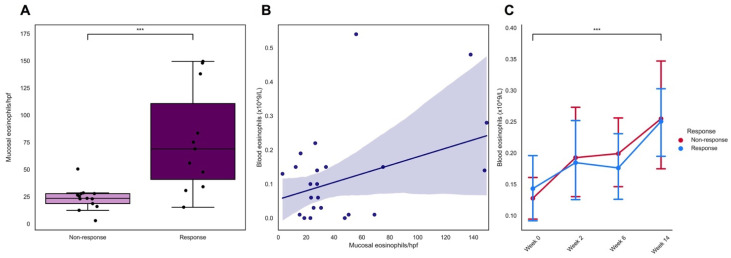
(**A**–**C**). Mucosal eosinophil abundance associates with clinical response to vedolizumab induction therapy in the discovery cohort. (**A**) Median eosinophils/hpf were significantly higher among patients who eventually responded to vedolizumab induction therapy vs. patients who showed no response. (**B**) Mucosal eosinophil counts were significantly associated to blood eosinophil concentrations. The blue line was fitted using a robust regression method by minimizing least trimmed squares (LTS), which is not unduly affected by outliers. The blue shade represents the 95% confidence interval (CI). (**C**) Blood eosinophil concentrations significantly increased after vedolizumab induction therapy. *** *p* < 0.001. Abbreviations: hpf, high power field.

From the total IBD cohort, we included histopathological data of twenty-four (24) patients with non-inflamed biopsies from the ascending colon. The median [IQR] eosinophil count in non-inflamed colonic tissue of these 24 patients was 28 [IQR: 23–66] eosinophils/hpf. In patients responding to vedolizumab induction therapy (*n* = 12), the median eosinophil count was significantly higher compared to non-responders (*n* = 12) (69 [34–138] vs. 24 [18–28] eosinophils/hpf, respectively) (Figure 1A). Inter-observer agreement on the number of mucosal eosinophils per hpf was excellent, showing an almost perfect association (*r* = 0.96, *p* < 0.001) (Figure S2). Mucosal eosinophil counts (per hpf) were significantly associated with corresponding blood eosinophil concentrations (*r* = 0.47, *p* < 0.05, Figure 1B). Furthermore, blood eosinophil concentrations significantly increased after vedolizumab induction therapy (week 0: median 0.09 [IQR: 0.03–0.21] × 10^9^/L; week 14: 0.22 [0.09–0.36] × 10^9^/L (*p* < 0.001, Figure 1C). Baseline blood eosinophil counts did not show a predictive value for response to vedolizumab induction therapy (Table 2).

### 3.3. The Eosinophil Chemoattractant Eotaxin-1 Is Associated with Response to Vedolizumab Induction Therapy

**Figure 2:**
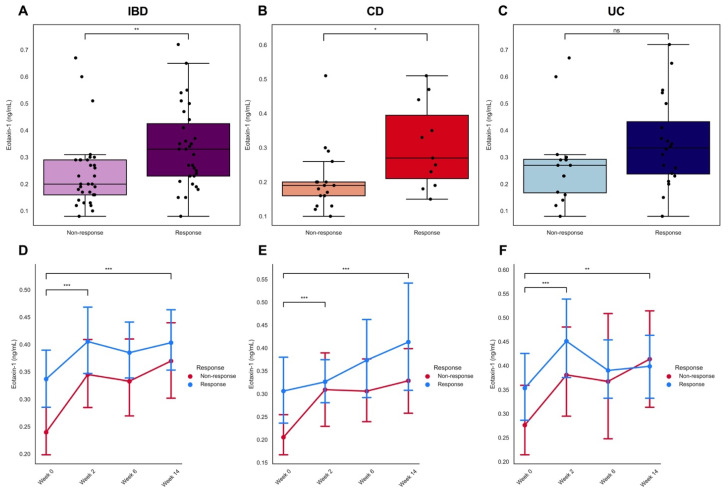
(**A**–**F**) Serum eotaxin-1 levels (ng/mL) among clinical responders and primary non-responders to vedolizumab induction therapy in the discovery cohort. (**A**) Baseline serum eotaxin-1 levels (ng/mL) were significantly higher in patients with IBD eventually responding to vedolizumab induction therapy versus those who did not. (**B**,**C**) Baseline serum eotaxin-1 levels (ng/mL) were significantly elevated in patients with Crohn’s disease (CD) who eventually clinically responded to vedolizumab, whereas in patients with UC, no significant difference was observed when comparing responders to non-responders. (**D**–**F**) Serum eotaxin-1 levels (ng/mL) significantly increased in patients during the course of vedolizumab induction therapy, in both responders and non-responders. * *p* < 0.05; ** *p* < 0.01; *** *p* < 0.001.

In the 64 patients with IBD in the discovery cohort, baseline serum eotaxin-1 levels were significantly higher in eventual responders to vedolizumab induction therapy as compared to primary non-responders (0.33 [0.23–0.44] vs. 0.20 [0.16–0.29] ng/mL, *p* < 0.01, Figure 2A). In patients with CD, serum eotaxin-1 levels were significantly elevated in responders (0.27 [0.19–0.44] vs. 0.19 [0.14–0.23] ng/mL, *p* < 0.05), whereas in patients with UC no significant difference was observed (0.33 [0.23–0.48] vs. 0.27 [0.16–0.30] ng/mL, *p* = 0.10, Figure 2C). Serum eotaxin-1 levels were not associated with mucosal eosinophil counts, although they were significantly inversely associated with blood eosinophil concentrations (Figures S3 and S4). After 2 weeks of vedolizumab induction therapy, serum eotaxin-1 levels significantly increased, both in CD and UC, irrespective of clinical response at week 14 (*p* < 0.01, Figure 2D–F). Moreover, serum eotaxin-1 levels further increased throughout the course of vedolizumab induction therapy, with increases in serum eotaxin-1 levels remaining significant at week 14 compared to baseline in all patients (all *p* < 0.01).

### 3.4. Identification of Predictors of Response to Vedolizumab Induction Therapy

In the subset of 24 patients with IBD of whom mucosal eosinophil data were obtained, univariable logistic regression analysis revealed that ^2^log-transformed mucosal eosinophil counts were significantly associated with an increased odds of clinical response or remission to vedolizumab induction therapy at week 14 (OR 9.59, 95% CI: 1.54–59.9). That is, each doubling of the number of mucosal eosinophils was significantly associated with a 9.59-fold increased odds of attaining clinical response or remission at week 14. In addition, in the subset of patients of whom serum eotaxin-1 levels were measured (*n* = 64), serum eotaxin-1 was significantly associated with an increased odds of clinical response or remission to vedolizumab induction therapy at week 14 (OR 2.99, 95% CI: 1.34–6.68) (Table 2). Furthermore, female sex (OR 0.35, 95% CI: 0.14–0.86) and ^2^log-transformed serum CRP levels at baseline (OR 0.75, 95% CI: 0.59–0.95) were significantly associated with a decreased odds of clinical response or remission to vedolizumab induction therapy at week 14. Among patients with CD, ^2^log-transformed mucosal eosinophil counts showed perfect separation between responders and non-responders, rendering calculation of logistic regression parameters impossible. In addition, serum eotaxin-1 levels were even more strongly associated with an increased odds of clinical response or remission to vedolizumab induction therapy at week 14 (OR 5.98, 95% CI: 1.24–28.8). Among patients with UC, the predictive value of both mucosal eosinophil counts and serum eotaxin-1 levels was lost, showing no significant predictions (OR 4.09, 95% CI: 0.88–19.0 and OR 1.98, 95% CI: 0.78–5.02, respectively).

Subsequently, a multivariable logistic regression model was composed using forced entry of covariates (Table 3). Covariate selection was based on results derived from univariable logistic regression analysis (sex) and factors considered to be clinically relevant (age, prior anti-TNF usage and co-medication). As such, in the final model (Model 3), all predictor-associated ORs were adjusted for age, sex, co-medication and prior anti-TNF therapy.

In multivariable logistic regression analysis using data of all patients with IBD, ^2^log-transformed serum eotaxin-1 levels (ng/mL) were still significantly associated with an increased odds of clinical response or remission to vedolizumab induction therapy at week 14 (Model 3; OR 2.87, 95% CI: 1.09–7.55). However, ^2^log-transformed serum CRP levels at baseline lost their significance in predicting vedolizumab induction therapy response (Model 3; OR 0.77, 95% CI: 0.60–1.01).

In the multivariable analyses stratified for diagnosis, no significant predictors for clinical response or remission were demonstrated, though, in patients with CD, ^2^log-transformed serum eotaxin-1 levels were still statistically significant after adjustment for age and sex (Model 2; OR 8.29, 95% CI: 1.23–55.9, *p* < 0.05).

### 3.5. Overall Classification Performance of Mucosal Eosinophil Abundance and Serum Eotaxin-1 Levels Regarding Clinical Response to Vedolizumab Induction Therapy

**Figure 3:**
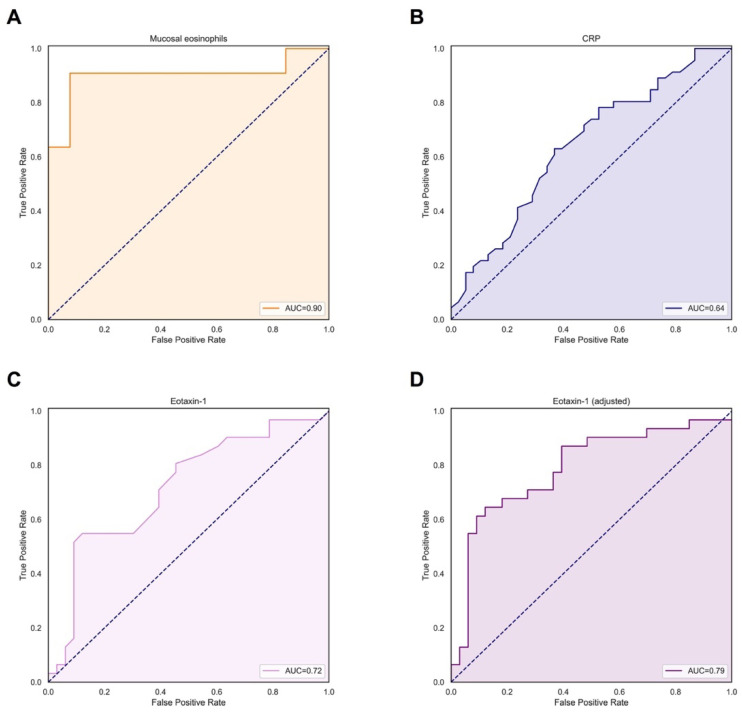
(**A**–**D**) Receiver operating characteristics (ROC) curves for mucosal eosinophil counts/hpf. (**A**) serum CRP levels (mg/L) (**B**) serum eotaxin-1 levels (ng/mL) (**C**) and adjusted serum eotaxin-1 levels (combined predicted probability of multivariable logistic regression model) (**D**) for discriminating responders and non-responders to vedolizumab among patients with IBD. The best discriminative performance to predict clinical response or remission to vedolizumab induction therapy was demonstrated by mucosal eosinophil counts (although based on a subset of *n* = 24 patients), while the performance of the multivariable model of serum eotaxin-1 levels (*n* = 64) also showed reasonable discrimination. Data presented are based on patients from the discovery cohort. Abbreviations: CRP, C-reactive protein; AUC, area under the ROC curve.

To further analyze the predictive accuracy of mucosal eosinophil abundance and serum eotaxin-1 levels with respect to clinical response or remission to vedolizumab induction therapy, receiver operating characteristics (ROC) analyses were performed. In the total IBD cohort, both mucosal eosinophil counts (*n* = 24) and serum eotaxin-1 levels (*n* = 64) significantly discriminated between patients who responded and did not respond to vedolizumab induction therapy, as represented by areas under the ROC curve (AUCs) of 0.90 (95% CI: 0.75–1.00, *p* < 0.01) and 0.72 (95% CI: 0.59–0.85, *p* < 0.01), respectively (Table 4 and Figure 3). In order to evaluate the goodness-of-fit of the multivariable model with serum eotaxin-1 levels as predictor, a combined predicted probability for achieving clinical response or remission to vedolizumab induction therapy was calculated, which yielded an AUC of 0.79 (95% CI: 0.67–0.91, *p* < 0.01) (Figure 3D). Both mucosal eosinophil abundance and serum eotaxin-1 levels were better predictors of response compared to serum CRP levels, the latter showing a lower discriminative performance (AUC 0.64 [0.52–0.76], *p* = 0.03). After *k*-fold cross-validation, discriminative performances of both mucosal eosinophils (cv-AUC = 0.90 [0.80–1.00]) and serum eotaxin-1 levels (cv-AUC: 0.74 [0.66–0.82]) were retained, although with greater uncertainty (as expected, when adjusting for model optimism).

Univariately, an optimal cut-off value of >0.31 ng/mL for serum eotaxin-1 levels had a sensitivity of 54.8% and a specificity of 87.9% in predicting clinical response or remission to vedolizumab induction therapy at week 14 (Youden’s *J* statistic: 0.43). Using the multivariable model, the same threshold of 0.31 ng/mL had a sensitivity of 64.5% and specificity of 87.9% in predicting clinical response (Youden’s *J* statistic: 0.52). Mucosal eosinophil counts had an optimal sensitivity and a specificity of 90.9% and 92.3%, respectively, with a corresponding cut-off value of >30 eosinophils/hpf (Youden’s *J* statistic 0.83). By comparison, the best cut-off value of <4.6 mg/L for serum CRP levels had a sensitivity of 63.2% and a specificity of 63.0% in predicting vedolizumab induction therapy response (Youden’s *J* statistic: 0.26).

### 3.6. External Validation of Serum Eotaxin-1 Levels as Predictor of Clinical Response to Vedolizumab Induction Therapy in the GEMINI Cohort

**Figure 4:**
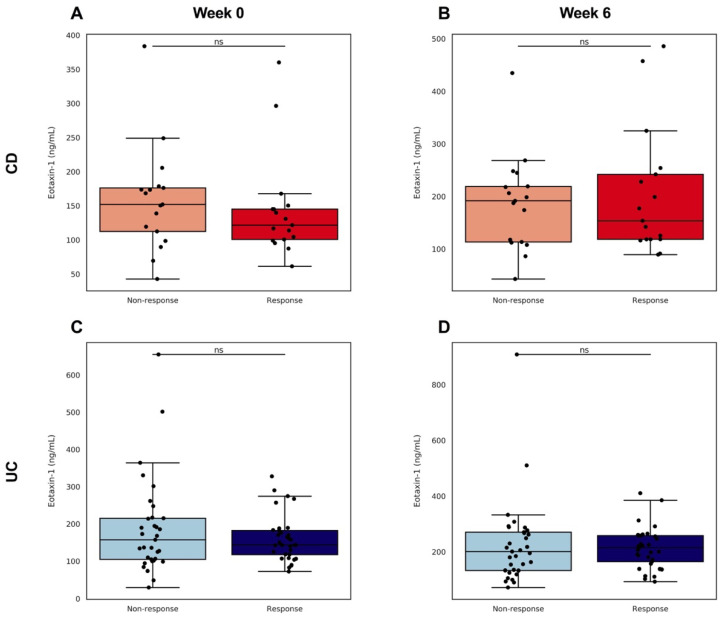
(**A**–**D**) Serum eotaxin-1 levels at week 0 and week 6in responders and non-responders to vedolizumab induction therapy in patients from the GEMINI cohort. (**A**,**B**) Patients with CD showed no differences in serum eotaxin-1 levels at baseline nor after six weeks of therapy. (**C**,**D**) Similarly, patients with UC demonstrated no significant differences at baseline or at week 6 of therapy.

Eotaxin-1 levels were quantified at week 0 and week 6 in 33 pairs of UC and 17 pairs of CD patients who did or did not respond to vedolizumab induction therapy (in a 1:1 ratio). Eotaxin-1 levels at each of these time points, as well as the fold changes in eotaxin-1, showed no significant differences between responders and non-responders. (Figure 4, Table S1).

Additionally, clinical response and remission during maintenance time points (weeks 14 and 52) were not associated with week 0 and week 6 eotaxin-1 concentrations using a logistic regression incorporating baseline CDAI or Mayo scores, age, sex, and maintenance treatment as covariates (Table S2). It is important to note that while all selected patients were treated with vedolizumab induction therapy in the GEMINI trials, responders at week 6 were randomized to the maintenance arm of the study.

## 4. Discussion

In the present study, we demonstrated that eosinophil abundance in non-inflamed parts of the colonic mucosa is associated with response to vedolizumab induction therapy in patients with IBD. A higher mucosal count in non-inflamed parts of colonic tissue was associated with therapy response. Additionally, we have shown that vedolizumab therapy significantly increases the amount of peripheral blood eosinophils at week 14. Despite the fact that the results from our validation cohort (an aggregate of matched responders and non-responders to vedolizumab therapy in the GEMINI I and II trials) did not confirm our initial findings, we showed that serum eotaxin-1 levels could accurately discriminate between responders and non-responders to vedolizumab induction treatment in the Dutch discovery cohort.

One of the main goals within IBD research is to establish highly effective treatment by predicting therapy response in individual patients. In the present Dutch real-life cohort of 84 patients with IBD, vedolizumab was effective for 38 patients (45%) at week 14. These efficacy results are consistent with other published real-world studies [10,13,14]. Multiple predictive factors for response to vedolizumab therapy in IBD have so far been investigated, including patient-related factors (e.g., age, BMI, smoking status), clinical factors (e.g., disease severity) and medication-related factors (e.g., concomitant use of immunomodulators) [27]. Frequently used measures of inflammatory disease activity, e.g., C-reactive protein (CRP) or the fecal calprotectin (FC) level, have been studied for their predictive performance. In general, patients with IBD treated with vedolizumab are less likely to respond in case of severe disease activity at baseline and prior failure to anti-TNF [27]. For instance, elevated baseline CRP concentrations are inversely related to vedolizumab therapy response or remission at week 14 [10].

In the present study, eosinophil abundance in non-inflamed parts of ascending colonic tissue was associated with an increased odds of responding to vedolizumab induction therapy. Although the exact underlying mechanism behind this observation remains elusive, we assume that this association might be based on the pharmacodynamic properties of vedolizumab. We hypothesized that the high eosinophil count in the non-inflamed colonic tissue may be a proxy reflecting that eosinophilic trafficking to the gut is accelerated in these patients. This could be due to the increased expression of several cell migration proteins on both eosinophils and endothelial cells. Patients who show this high eosinophilic migration pattern might benefit more from blocking the α4β7 integrin, resulting in a response to vedolizumab treatment. Instead, non-responders to vedolizumab treatment, showing a lower number of mucosal eosinophils, might have an inflamed reaction to the tissue caused by other factors.

The correlation between mucosal eosinophil counts and response to vedolizumab treatment after 6 months has been reported before [21]. In contrast to our findings, however, that study showed that higher eosinophil counts were associated with vedolizumab treatment *failure* instead of treatment *response* in patients with ulcerative colitis, and a similar trend was observed in patients with Crohn’s disease. Several reasons may explain these apparently contradictory results. First, Kim et al. determined the number of eosinophils in inflamed intestinal tissue, whereas we quantified the number of eosinophils in non-inflamed parts of colonic tissue. In circumstances of histopathological inflammation, quantification of eosinophils may be very difficult due to the dramatically increased number of those cells in inflamed tissue. Second, Kim et al. quantified eosinophils in both ileal and colonic tissue without specifying the sublocation in the colon, while it is known that eosinophilic counts may considerably differ across different anatomical locations in the intestines [24]. Therefore, we have specifically decided to only include intestinal tissue that was sampled from the non-inflamed ascending colon to avoid any differences that could be attributed to location. Third, Kim et al. defined response to vedolizumab treatment as between 14 and 30 weeks, whereas we investigated associations with therapy response at week 14, which is regarded as the evaluation time point for response to induction therapy [7,8,10]. As such, patients could have lost vedolizumab treatment response or experienced response to the treatment between week 14 and week 30.

For now, we can only speculate about the discrepancy between the two cohorts included in this study regarding serum eotaxin-1 levels as a potentially predictive biomarker for response to vedolizumab induction therapy. For example, the majority of patients included in the UMCG discovery cohort were Dutch Caucasians from a rather small region referred to one tertiary center, whereas the GEMINI validation cohort was characterized by a rather mixed ethnic composition with patients that were included from different geographical locations. Such geographic and ethnic differences may have impacted eosinophil and/or eotaxin-1 biology, e.g., through differing environmental effects and potential genetic susceptibility. However, results from the validation cohort of the GEMINI 1 and GEMINI 2 studies did show a trend towards higher serum eotaxin-1 levels after six weeks of therapy in both CD and UC, which was also observed (significantly) in the Dutch cohort [13,14,28].

To the best of our knowledge, the utility of serum eotaxin-1 levels to predict response to vedolizumab therapy in IBD has not yet been evaluated. Eotaxin-1 (CCL11) is a selective chemoattractant that activates and mobilizes eosinophils to the lamina propria of the gut [29,30]. Eosinophils are established as key regulators in the initiation and progression of the inflammatory response, causing intestinal tissue damage and dysfunction [31]. In active IBD, the intestinal mucosa is highly populated with active eosinophils [30,32,33]. Elevated eotaxin-1 levels in conjunction with increased levels of serum eosinophils have been demonstrated in patients with active IBD, including the correlation with disease severity, and most prominently in CD [34,35,36]. In our study, we observed relatively higher levels of serum eotaxin-1 and higher numbers of mucosal eosinophils in the non-inflamed parts of the biopsies of patients with CD who eventually responded to vedolizumab induction therapy in comparison to non-responders, both of which also demonstrated more pronounced discriminative accuracy regarding clinical response. In this relatively small cohort, we could not show a significant difference in baseline eotaxin-1 levels in UC patients responding to vedolizumab treatment, although we did observe a clear trend towards increased baseline eotaxin-1 levels in responders compared to non-responders. Eotaxin-1 is overexpressed in IBD and is most prominently observed in patients with active disease compared to quiescent disease stages [37]. The above-described hypotheses on the underlying pathophysiological mechanism(s) that might be involved in the eosinophil-eotaxin-1 signaling may be more important in the context of vedolizumab treatment for patients with CD compared to UC. Eotaxin-1 is mainly produced by intestinal epithelial cells, endothelial cells and macrophages and is influenced by other cytokines that correlate well with IBD disease activity [38,39]. Furthermore, serum eotaxin-1 levels can distinguish quiescent from active IBD both in human and experimental colitis models, further substantiating its role in disease pathogenesis [36,40,41].

Our study has several strengths. Most notably, in our study cohort, we observed two potentially powerful predictors of response to vedolizumab induction therapy, also when adjusted for potential confounding factors (age, gender, combination therapy, prior anti-TNF therapy). We strictly selected only non-inflamed colonic ascending tissue to correct for any variance in eosinophil counts caused by inflammation or location in the gut and all biopsies were taken within a window of three months prior to the start of vedolizumab treatment. All histopathological data were assessed by two trained researchers. Another important strength of our study consisted of the highly sensitive, rigorously validated electrochemiluminescence (ECL) assay that was used for measuring serum eotaxin-1 concentrations. We performed external validation of our eotaxin-1 results using data derived from probably the best-described and best-documented cohorts on vedolizumab treatment: the GEMINI trials. Although these results did not confirm our initial findings, we still consider this validation attempt of the results on eotaxin-1 in our discovery cohort as an important methodological strength.

However, some major limitations of this study also must be considered. Most importantly, because of its retrospective origin, the data collection was not optimal as we could only count mucosal eosinophils in a small subset (29%) of patients of whom biopsies were available from non-inflamed colonic regions. Similarly, we had no structural endoscopic follow-up or fecal calprotectin levels available after induction therapy, which impeded us from objectifying our clinical disease activity data and mucosal eosinophil counts in the gut mucosa after therapy. Another limitation is that our primary study outcome was based on validated clinical disease activity indices (HBI/SCCAI) that were recorded and documented by different certified gastroenterologists or specialized IBD nurse practitioners. Still, these scoring methods remain prone to inter-individual differences in perception and interpretation of subjective patient information. Finally, a greater sample size might have allowed us to identify more significant predictors of response to vedolizumab therapy and reproduce more reliable subgroup analyses for patients with CD and UC separately.

## 5. Conclusions

In this pilot study, we demonstrated a strong correlation between mucosal eosinophil abundance in non-inflamed ascending colonic tissue and response to vedolizumab treatment in patients with IBD. Further, there might be a correlation between baseline eotaxin-1 levels and vedolizumab treatment response in specific/local and more homogeneous patient cohorts, despite the conflicting results from the external validation data. Collecting biopsies from non-inflamed colonic areas could be integrated into clinical practice as well as quantification of eosinophils. Larger prospective cohorts including endoscopic follow-up, fecal calprotectin levels and a reliable combination of inflammatory biomarkers are however warranted to verify the presented results.

## Figures and Tables

**Table 1:** Baseline cohort demographic and clinical characteristics of patients with IBD within the discovery cohort receiving vedolizumab induction therapy, stratified by clinical response at week 14.

IBD	Response	Non-Response	*p*-Value ^‡^
	*n* = 38	*n* = 46	
Age (years)	43.6 ± 15.6	43.3 ± 15.3	0.95
Female sex, *n* (%)	16 (42.1)	31 (67.4)	0.02
BMI (kg/m^2^) ^†^	23.4 [20.5;27.1]	24.2 [21.3;28.0]	0.35
IBD diagnosis			0.10
*CD*, *n* (%)	13 (34.2)	24 (52.2)	
*UC*, *n* (%)	25 (65.8)	22 (47.8)	
Smoking status			0.33
*Never*, *n* (%)	24 (63.2)	22 (47.8)	
*Former*, *n* (%)	7 (18.4)	14 (30.4)	
*Current*, *n* (%)	7 (18.4)	10 (21.7)	
Prior anti-TNF usage *			0.58
*None*, *n* (%)	3 (7.9)	4 (8.7)	
*One*, *n* (%)	21 (55.3)	19 (41.3)	
*Two*, *n* (%)	12 (31.6)	18 (39.1)	
*Three*, *n* (%)	2 (5.3)	5 (10.9)	
**Montreal Age (A)**			0.86
A1 (≤16 years), *n* (%)	5 (13.2)	8 (17.4)	
A2 (17–40 years), *n* (%)	24 (63.2)	27 (58.7)	
A3 (>40 years), *n* (%)	9 (23.7)	11 (23.9)	
**Montreal Location (L, CD)**			0.12
L1 (ileal), *n* (%)	2 (15.4)	2 (8.3)	
L2 (colonic), *n* (%)	0 (0.0)	4 (16.7)	
L3 (ileocolonic), *n* (%)	9 (69.2)	17 (70.8)	
L1 + L4 (upper GI), *n* (%)	0 (0.0)	1 (4.2)	
L2 + L4 (upper GI), *n* (%)	0 (0.0)	0 (0.0)	
L3 + L4 (upper GI), *n* (%)	2 (15.4)	0 (0.0)	
**Montreal Behavior (B, CD)**			0.75
B1 (non-stricturing, non-penetrating), *n* (%)	5 (38.5)	10 (41.7)	
B2 (stricturing), *n* (%)	6 (46.2)	8 (33.3)	
B3 (penetrating), *n* (%)	2 (15.4)	6 (25.0)	
**Montreal Perianal disease (*p*, CD)**			0.30
No, *n* (%)	10 (76.9)	15 (62.5)	
Yes, *n* (%)	3 (23.1)	9 (37.5)	
**Montreal Extension (E, UC)**			0.52
E1 (proctitis)	0 (0.0)	0 (0.0)	
E2 (left-sided colitis)	6 (24.0)	8 (36.4)	
E3 (pancolitis)	19 (76.0)	14 (63.6)	
**Montreal Severity (S, UC)**			0.58
S1 (mild)	2 (8.0)	3 (13.6)	
S2 (moderate)	12 (48.0)	13 (59.1)	
S3 (severe)	11 (44.0)	6 (27.3)	
**Concomitant Medication**			
None, *n* (%)	17 (44.7)	15 (32.6)	0.27
Aminosalicylates, *n* (%)	12 (31.6)	11 (23.9)	0.47
Thiopurines/MTX, *n* (%)	5 (13.2)	13 (28.3)	0.11
Steroids, *n* (%)	2 (5.3)	3 (6.5)	1.00
Combination therapy, *n* (%)	2 (5.3)	4 (8.7)	0.69
**Laboratory Parameters**			
Hemoglobin (mmol/L)	7.6 ± 1.3	7.4 ± 1.0	0.41
CRP (mg/L) ^†^	3.4 [1.3;9,5]	7.0 [3.2;16.8]	0.03
ESR (mm/h) ^†^	22 [7;45]	23 [10;46]	0.41
WBC (×10^9^/L) ^†^	8.0 [6.3;10.0]	7.6 [6.0;10.6]	0.69
Thrombocytes (×10^9^/L) ^†^	309 [255;386]	335 [288;392]	0.25
Eosinophils (×10^9^/L) ^†^	0.07 [0.01;0.26]	0.10 [0.04;0.20]	0.64
**Clinical Disease Activity ^§^**			
HBI (CD)			1.00
*Remission* (<5), *n* (%)	1 (8.3)	2 (9.5)	
*Active disease* (≥5), *n* (%)	11 (91.7)	19 (90.5)	
SCCAI (UC)			1.00
*Remission* (≤2), *n* (%)	1 (5.3)	1 (5.0)	
*Active disease* (>2), *n* (%)	18 (94.7)	19 (95.0)	
**Surgical History**			
Ileocecal resection, *n* (%)	8 (21.1)	13 (28.3)	0.61
Colectomy, *n* (%)	0 (0.0)	1 (2.2)	1.00

Data are presented as mean ± SD or proportions with corresponding percentages (*n*, %). * Prior anti-TNF usage included the use of the biologicals infliximab, adalimumab, and golimumab. ^†^ Skewed variables are presented as median [IQR]. Differences between groups were tested according to normality using independent sample *t*-tests or Mann–Whitney *U*-tests for continuous variables and chi-square tests or Fisher’s exact tests for categorical variables, as appropriate. ^‡^ Two-sided *p*-values < 0.05 were considered statistically significant. ^§^ HBI scores were available for *n* = 33 patients with CD (89.2%), and SCCAI scores were available for *n* = 39 patients with UC (82.9%). Abbreviations: IBD, inflammatory bowel disease; CD, Crohn’s disease; UC, ulcerative colitis; BMI, body mass index; MTX, methotrexate; HBI, Harvey-Bradshaw index; SCCAI, simple clinical colitis activity index; CRP, C-reactive protein; ESR, erythrocyte sedimentation rate; WBC, white blood cell count.

**Table 2:** Univariable logistic regression analysis of predictors of clinical response or remission to vedolizumab induction therapy at week 14 in patients with IBD included in the discovery cohort.

	IBD (*n* = 84)		CD (*n* = 37)		UC (*n* = 47)	
	OR	95% CI	OR	95% CI	OR	95% CI
Age (years)	1.00	0.97–1.03	1.01	0.97–1.05	1.00	0.96–1.03
Female sex	0.35 *	0.14–0.86	0.66	0.16–2.73	0.27 *	0.08–0.90
BMI (kg/m^2^)	0.97	0.89–1.07	0.93	0.78–1.11	0.97	0.86–1.09
Active smoking (yes/no)	0.64	0.21–1.98	0.94	0.21–4.29	0.72	0.09–5.81
Co-medication (yes/no)	0.60	0.25–1.45	0.38	0.09–1.51	0.67	0.19–2.31
Prior anti-TNF therapy	1.11	0.23–5.30	0.52	0.03–9.10	1.82	0.27–12.0
Prior surgery (yes/no)	0.61	0.22–1.66	1.35	0.34–5.36	^§^	^§^
Clinically active disease vs. remission (HBI/SCCAI)	1.15	0.18–7.30	1.16	0.09–14.3	0.95	0.06–16.3
Baseline CRP (mg/L) *	0.75 *	0.59–0.95	0.69	0.47–1.03	0.84	0.61–1.14
Baseline Eotaxin-1 (ng/mL) *	2.99 *	1.34–6.68	5.98 *	1.24–28.8	1.98	0.78–5.02
Baseline mucosal eosinophils/hpf *	9.59 *	1.54–59.9	^‡^	^‡^	4.09	0.88–19.0
Baseline blood eosinophils *	0.90	0.73–1.12	1.03	0.69–1.56	0.88	0.67–1.14

Data are presented as odds ratios (OR) with corresponding 95% confidence intervals (CI) derived from univariable logistic regression models without additional covariates (model 1, crude). * *p*-value < 0.05. ^‡^ Perfect separation between groups, no calculations possible. ^§^ Only one case, no calculations possible.

**Table 3:** Multivariable logistic regression analysis of serum CRP, eotaxin-1 and mucosal eosinophil counts as predictors of clinical response or remission to vedolizumab induction therapy at week 14 of patients from the discovery cohort. Adjusted odds ratios (ORs) (with 95% CI) were calculated for all IBD patients, and for CD and UC cohorts separately.

		IBD (*n* = 84)		CD (*n* = 37)		UC (*n* = 47)	
*Predictors ^†^*	Model	OR	95% CI	OR	95% CI	OR	95% CI
CRP (mg/L)	1	0.75 *	0.59–0.95	0.69	0.47–1.03	0.84	0.61–1.14
	2	0.79	0.62–1.02	-	-	-	-
	3	0.77	0.60–1.01	-	-	-	-
Mucosal eosinophils/hpf	1	9.59 *	1.54–59.9	¶	¶	¶	¶
	2	¶	¶	¶	¶	¶	¶
	3	¶	¶	¶	¶	¶	¶
Eotaxin-1 (ng/mL)	1	2.99 *	1.34–6.68	5.98 *	1.24–28.8	1.98	0.78–5.02
	2	2.49 *	1.03–6.00	8.29 *	1.23–55.9	-	-
	3	2.87 *	1.09–7.55	¶	¶	-	-

Data are presented as odds ratios (OR) with corresponding 95% confidence intervals (CI) derived from univariable (Model 1) and multivariable (Model 2 and 3) logistic regression models. ^† 2^log-transformed predictor variables. Model 1: univariable logistic regression analysis, see also Table 2. Model 2: model 1, additionally adjusted for age and sex. Model 3: model 2, additionally adjusted for combination therapy and history of prior anti-TNF therapy. * *p*-value < 0.05. ¶ Event-per-predictor (EPV) variable ratio < 10, no multivariable analysis performed.

**Table 4:** Receiver operating characteristics (ROC) analysis demonstrating discriminative values of mucosal eosinophil abundance, serum eotaxin-1 levels (unadjusted and adjusted models), and serum CRP levels with regard to clinical response to vedolizumab induction therapy. Data presented in this table are based on patients from the discovery cohort.

	AUC (95% CI)	CV-AUC (95% CI)	*p*-Value	Sensitivity	Specificity	Optimal Cut-Off	Youden’s *J* Statistic
***IBD***							
CRP	0.64 (0.52–0.76)	0.66 (0.59–0.73)	0.03	63.2%	63.0%	<4.6 mg/L	0.26
Eotaxin-1	0.72 (0.59–0.85)	0.74 (0.66–0.82)	<0.01	54.8%	87.9%	>0.31 ng/mL	0.43
Eotaxin-1 (adjusted)	0.79 (0.67–0.91)	0.81 (0.76–0.86)	<0.01	64.5%	87.9%	-	0.52
Mucosal eosinophils	0.90 (0.75–1.00)	0.90 (0.80–1.00)	<0.01	90.9%	92.3%	>30/hpf	0.83
***CD***							
CRP	0.70 (0.52–0.88)	0.64 (0.53–0.75)	0.05	53.8%	87.5%	<3.4 mg/L	0.41
Eotaxin-1	0.75 (0.57–0.94)	0.80 (0.79–0.91)	0.03	72.7%	52.7%	>0.22 ng/mL	0.49
Eotaxin-1 (adjusted)	0.73 (0.53–0.93)	0.65 (0.50–0.80)	0.05	45.5%	100%	-	0.46
Mucosal eosinophils	^†^	^†^	^†^	^†^	^†^	^†^	^†^
***UC***							
CRP	0.57 (0.40–0.73)	0.50 (0.42–0.58)	0.45	28.0%	90.9%	<1.1 mg/L	0.19
Eotaxin-1	0.66 (0.48–0.84)	0.60 (0.48–0.72)	0.10	55.0%	87.5%	>0.32 ng/mL	0.43
Mucosal eosinophils	^†^	^†^	^†^	^†^	^†^	^†^	^†^

^†^ Too few events, no discrimination analysis performed. Abbreviations: AUC, area under the curve; CD, Crohn’s disease; CRP, C-reactive protein; CV-AUC, cross-validated area under the curve; IBD, inflammatory bowel disease; UC, ulcerative colitis.

## Data Availability

The datasets used and/or analyzed for the current study are available from the corresponding author upon reasonable request.

## Supplementary Materials

The following supporting information can be downloaded at: https://www.mdpi.com/article/10.3390/jcm11144141/s1, Table S1: *p*-values of Wilcoxon signed-rank tests comparing eotaxin-1 levels at week 0, week 6 and fold changes from week 0 to week 6, in matched pairs of UC and CD patients responding or not responding to vedolizumab induction therapy. Table S2: Clinical response and remission during maintenance timepoints (weeks 14 and 52) were associated with week 0 and week 6 eotaxin-1 concentrations using a logistic regression incorporating baseline CDAI or Mayo scores, age, sex and maintenance treatment as covariates. The table below shows the odds ratio associated with a 1 ng/mL increase in eotaxin-1, the 95% confidence interval of this estimate and *p*-values from a likelihood ratio test comparing models with and without the eotaxin-1 term. Figure S1: Overview of the study design. Figure S2: Inter-observer agreement on the abundance of mucosal eosinophils in pre-treatment intestinal biopsies from non-inflamed regions in the ascending colon. Figure S3: Mucosal eosinophil abundance was not associated to serum eotaxin-1 levels in patients with IBD. Figure S4: Blood eosinophil concentrations (×10^9^/L) were significantly inversely associated with serum eotaxin-1 levels (ng/mL).
