# Supplementary material for: Mucosal Eosinophil Abundance in Non-Inflamed Colonic Tissue Is Associated with Response to Vedolizumab Induction Therapy in Inflammatory Bowel Disease
Source: J Clin Med. 2022 Jul 16;11(14):4141. doi: 10.3390/jcm11144141 (PMC9318498; doi:10.3390/jcm11144141)
Supplement: Supplementary file 1 [file jcm-11-04141-s001.zip › jcm-1787260-supplementary.pdf]

Supporting Information

Supplementary Table S1. *P*-values of Wilcoxon signed-rank tests comparing eotaxin-1 levels at week 0, week 6, and fold changes from week 0 to week 6, in matched pairs of UC and CD patients responding or not responding to vedolizumab induction therapy.

Timepoint	Ulcerative colitis	Crohn's Disease
Week 0	0.480	0.263
Week 6	0.930	0.927
Fold change	0.416	0.353

Supplementary Table S2. Clinical response and remission during maintenance timepoints (weeks 14 and 52) were associated with week 0 and week 6 eotaxin-1 concentrations using a logistic regression incorporating baseline CDAI or Mayo scores, age, sex, and maintenance treatment as covariates. The table below shows the odds ratio associated with a 1 ng/ml increase in eotaxin-1, the 95% confidence interval of this estimate and *P*-values from a likelihood ratio test comparing models with and without the eotaxin-1 term.

Eotaxin-1	Outcome	Ulcerative Colitis	Crohn's disease
Week 0	Week 14 Clinical Response	1 [0.99-1] (0.615)	1 [0.99-1] (0.835)
	Week 52 Clinical Response	0.9 [0-Inf] (1)	1.3 [0-Inf] (1)
	Week 52 Clinical Remission	1 [0.97-1.1] (0.490)	1 [0-Inf] (1)
Week 6	Week 14 Clinical Response	1 [0.99-1] (0.938)	1 [0.99-1] (0.634)
	Week 52 Clinical Response	1.2 [0-Inf] (1)	0.84 [0-Inf] (1)
	Week 52 Clinical Remission	0.99 [0.96-1] (0.658)	0.29 [0-Inf] (1)

Supplementary Figure S1

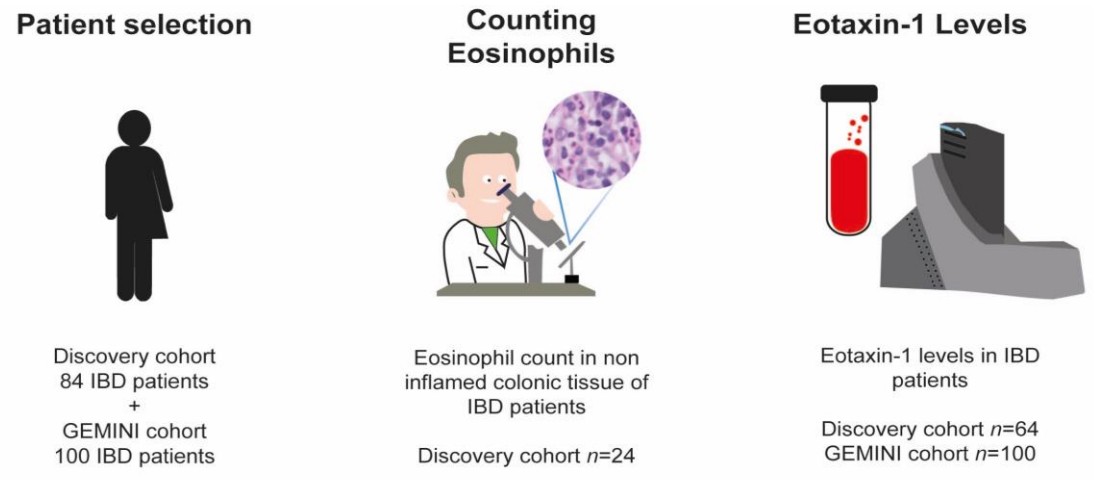

Supplementary Figure S1. Overview of the study design.

Supplementary Figure S2

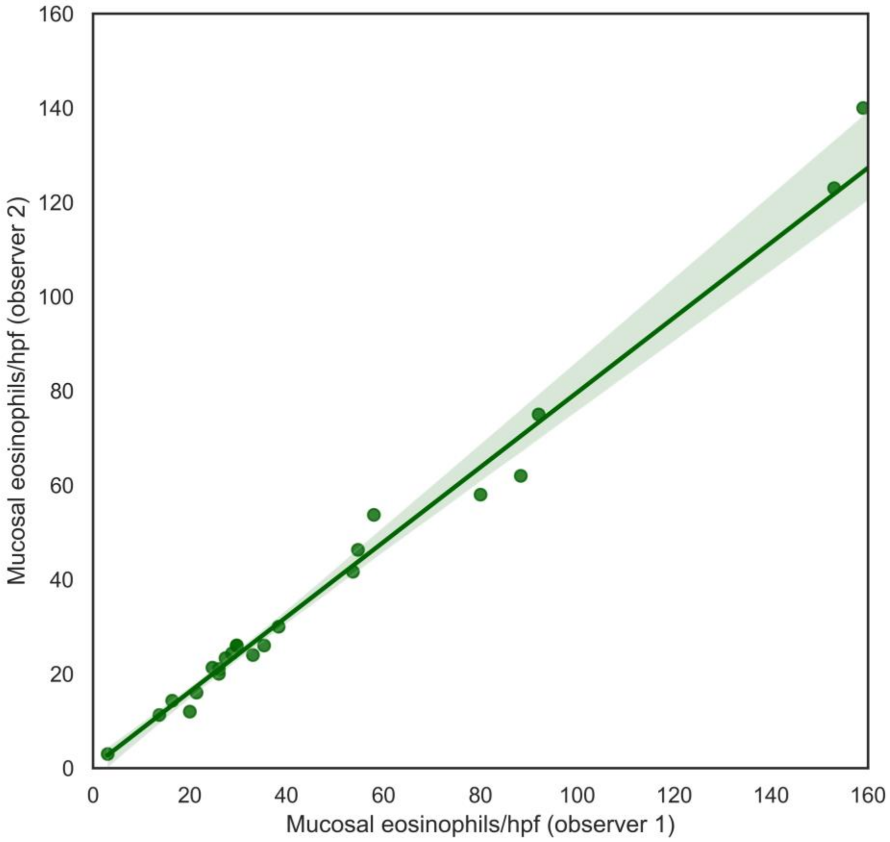

Supplementary Figure S2. Inter-observer agreement on the abundance of mucosal eosinophils in pre-treatment intestinal biopsies from non-inflamed regions in the colon ascendens.

Supplementary Figure S3

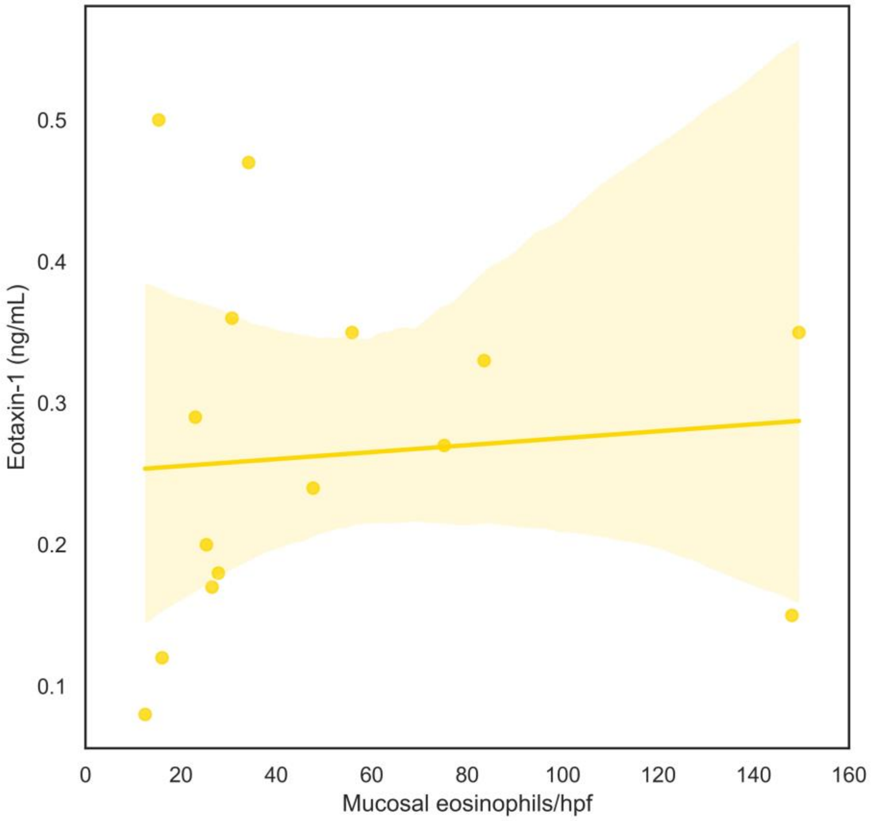

Supplementary Figure S3. Mucosal eosinophil abundance was not associated to serum eotaxin-1 levels in patients with IBD.

Supplementary Figure S4

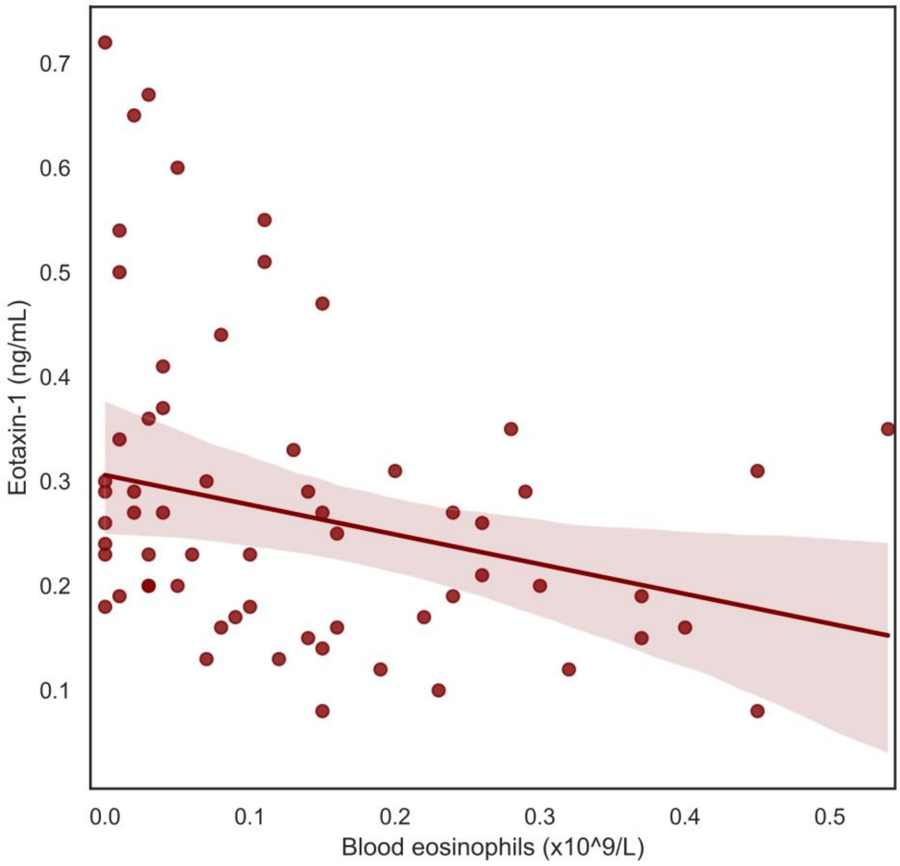

Supplementary Figure S4. Blood eosinophil concentrations (x10^9/L) were significantly inversely associated with serum eotaxin-1 levels (ng/mL).
